# Asymmetric high-order anatomical brain connectivity sculpts effective connectivity

**DOI:** 10.1162/netn_a_00150

**Published:** 2020-09-01

**Authors:** Arseny A. Sokolov, Peter Zeidman, Adeel Razi, Michael Erb, Philippe Ryvlin, Marina A. Pavlova, Karl J. Friston

**Affiliations:** Wellcome Centre for Human Neuroimaging, Institute of Neurology, University College London, London, United Kingdom; Department of Neurology, University Neurorehabilitation, University Hospital Inselspital, University of Bern, Bern, Switzerland; Service de Neurologie and Neuroscape@NeuroTech Platform, Département des Neurosciences Cliniques, Centre Hospitalier Universitaire Vaudois, Lausanne, Switzerland; Neuroscape Center, Weill Institute for Neurosciences, Department of Neurology, University of California San Francisco, San Francisco, CA, USA; Wellcome Centre for Human Neuroimaging, Institute of Neurology, University College London, London, United Kingdom; Wellcome Centre for Human Neuroimaging, Institute of Neurology, University College London, London, United Kingdom; Monash Institute of Cognitive and Clinical Neurosciences & Monash Biomedical Imaging, Monash University, Clayton, Australia; Department of Electronic Engineering, NED University of Engineering and Technology, Karachi, Pakistan; Department of Biomedical Magnetic Resonance, University of Tübingen Medical School, Tübingen, Germany; Service de Neurologie and Neuroscape@NeuroTech Platform, Département des Neurosciences Cliniques, Centre Hospitalier Universitaire Vaudois, Lausanne, Switzerland; Department of Psychiatry and Psychotherapy, University of Tübingen Medical School, Tübingen, Germany; Wellcome Centre for Human Neuroimaging, Institute of Neurology, University College London, London, United Kingdom

**Keywords:** Effective connectivity, Structural connectivity, Network diffusion, Graph Laplacian

## Abstract

Bridging the gap between symmetric, direct white matter brain connectivity and neural dynamics that are often asymmetric and polysynaptic may offer insights into brain architecture, but this remains an unresolved challenge in neuroscience. Here, we used the graph Laplacian matrix to simulate symmetric and asymmetric high-order diffusion processes akin to particles spreading through white matter pathways. The simulated indirect structural connectivity outperformed direct as well as absent anatomical information in sculpting effective connectivity, a measure of causal and directed brain dynamics. Crucially, an asymmetric diffusion process determined by the sensitivity of the network nodes to their afferents best predicted effective connectivity. The outcome is consistent with brain regions adapting to maintain their sensitivity to inputs within a dynamic range. Asymmetric network communication models offer a promising perspective for understanding the relationship between structural and functional brain connectomes, both in normalcy and neuropsychiatric conditions.

## Author Summary

Measures of white matter connectivity can usefully inform models of causal and directed brain communication (i.e., effective connectivity). However, due to the inherent differences in biophysical correlates, recording techniques and analytic approaches, the relationship between anatomical and effective brain connectivity is complex and not fully understood. In this study, we use simulation of heat diffusion constrained by the anatomical connectivity of the network to model polysynaptic (high-order) anatomical connectivity. The outcomes afford more useful constraints on effective connectivity than conventional, typically monosynaptic white matter connectivity. Furthermore, asymmetric network diffusion best predicts effective connectivity. In conclusion, the data provide insights into how anatomical connectomes give rise to asymmetric neuronal message passing and brain communication.

## INTRODUCTION

**Figure 1.**
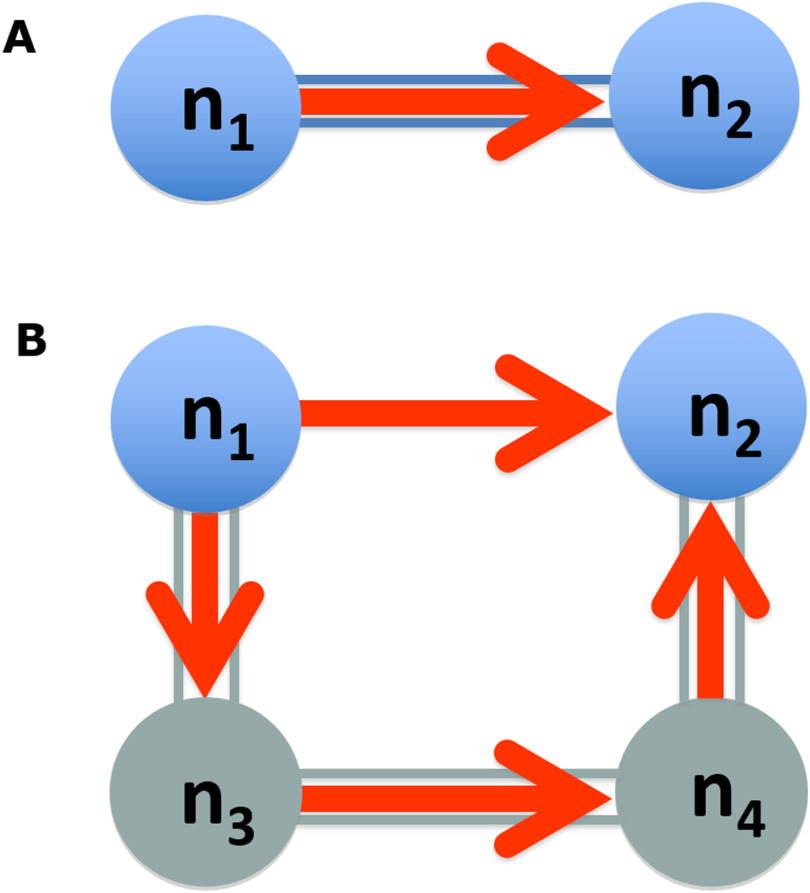
Illustration of the relationship between anatomical and effective brain connectivity. (A) Two network nodes n_1_ and n_2_ can have a structural pathway connecting them (blue double line) that may underlie causal functional influence of network node n_1_ over n_2_ (effective connectivity; orange arrow). (B) Effective connectivity from n_1_ to n_2_ may also be present in the absence of direct structural connectivity, mediated by polysynaptic structural (grey double lines) and effective connections through hidden nodes n_3_ and n_4_ (not specified in the network model). Modelling of indirect structural connectivity may therefore provide better constraints on effective connectivity than measures of direct structural connectivity alone.

Multimodal neuroimaging analyses are expected to improve our understanding of structure-function relationships in the brain (Toga et al., 2006; Honey et al., 2010; Sporns, 2014); drawing on measures of structural, functional, and effective brain connectivity (Sporns et al., 2000; Park & Friston, 2013). However, relating symmetric and static structural connectivity derived from diffusion magnetic resonance imaging (dMRI) to time-varying and context-sensitive functional dynamics (recorded by functional magnetic resonance imaging, fMRI, electroencephalography, EEG, or magnetoencephalography, MEG) remains an unresolved technical and conceptual challenge (Honey et al., 2009; Stephan et al., 2009; Pineda-Pardo et al., 2014; Uludag & Roebroeck, 2014). White matter (WM) pathways are sufficient for communication between brain regions, but functional brain dynamics can also be mediated through polysynaptic connections (Figure 1). Indeed, previous studies suggested the direct structural pathways inferred using dMRI account for only about 55% of measured resting-state functional connectivity patterns (Koch et al., 2002; Honey et al., 2009; Deligianni et al., 2011; Becker et al., 2016).

Current measures of anatomical and of resting-state functional connectivity are symmetric in the sense that they do not enable an assessment of whether one orientation of a pathway may be more prominent than the inverse (Friston, 2011). In contrast, models of effective connectivity such as dynamic causal models (DCMs) indicate the weights of specific directions of interaction (Friston et al., 2003), and recent data across species suggest that information about directed, asymmetric connectivity may more appropriately reflect brain architecture (Kale et al., 2018; Avena-Koenigsberger et al., 2019; Seguin et al., 2019).

Previous work has analysed the relationships between indirect anatomical connectivity and resting-state functional connectivity (Honey et al., 2007; Deligianni et al., 2011; Abdelnour et al., 2014; Becker et al., 2016; Meier et al., 2016; Bettinardi et al., 2017; Liang & Wang, 2017; Abdelnour et al., 2018). Recent graph-theoretic research has demonstrated that conventional, symmetric measures of brain WM architecture contain information on the differential efficiency of afferent and efferent network communication (Avena-Koenigsberger et al., 2019; Seguin et al., 2019). Furthermore, asymmetries in predicted communication efficiency were found to reflect neurobiological concepts of functional hierarchy and were correlated with directionality in resting-state effective connectivity analysed using spectral dynamic causal modelling (Seguin et al., 2019). Thus far, formal integration of effective with anatomical connectivity has only been implemented for direct and symmetric measures of structural connectivity (Stephan et al., 2009; Sokolov et al., 2018; Sokolov et al., 2019). The primary motivation for this study was to develop an integrative approach simulating symmetric and asymmetric high-order (polysynaptic) structural connectivity and using the outcomes to constrain models of task-related effective connectivity.

**Figure 2.**
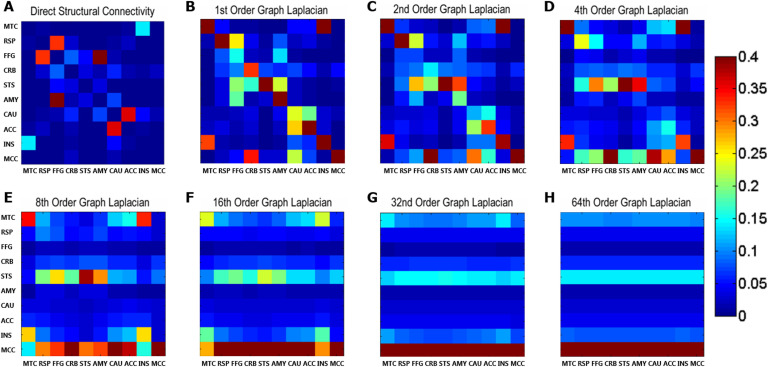
Network diffusion simulated by exponentiation of the matrix exponential of the graph Laplacian. (A) Based on the direct structural adjacency matrix derived from probabilistic tractography, using Equations 1–4, we created (B) the matrix exponential of the graph Laplacian (here, normalised to the in-degree). Exponentiation of the matrix exponential of the graph Laplacian simulated diffusion of particles constrained by the anatomical network and therefore yielded indirect structural connectivity. The 0th order is simply the identity matrix, meaning that each node (square on the diagonal) is equipped with an equal number of particles (spikes). Each increase in order corresponds to an additional propagation down another path or edge, and a subsequent distribution of spikes. The (first-order) matrix exponential thus represents the first small time step of diffusion. (C–G) As time (order) progresses, some nodes receive more particles (input) than others, until this closed system reaches a state of saturation (equilibrium), corresponding to the principle eigenmode of the graph Laplacian. (H) This eigenmode is visible at the 64^th^ order (time step of diffusion). The bands reflect how many spikes every node has received (in-degree of each node) and approximate the principle eigenvectors (Figure 4B). Please see Table 1 for abbreviated region labels.

This central aim inspired the use of the graph Laplacian (GL: see Materials and Methods) to compute polysynaptic symmetric and asymmetric structural connectivity. The GL is a construct from spectral graph theory and represents the difference between the adjacency (indicating which network nodes are interconnected) and degree matrices (indicating the number of nodes connected with each node). The GL can be used to simulate the diffusion of a conserved quantity of particles over the network (Figure 2 and Supporting Information Video S1; Biggs, 1993). Crucially, the GL approach allows introducing asymmetry (see Materials and Methods and Supporting Information Figure S1): weighting (normalising) the structural adjacency matrix to the in-degree implies that each target node has a fixed capacity to be influenced by other nodes, and its relative sensitivity is determined by the probability of receiving inputs. Conversely, if we normalise to the out-degree, we assume each node has a fixed capacity to influence other nodes, and the relative influence is proportional to efferent particle diffusion.

In our implementation, the GL matrix *L* is exponentiated and raised to the order *τ*, *exp*(*L*)^*τ*^. At the start of the diffusion process, each node is equipped with a large number of “spikes.” Each order *τ* represents a time step of the diffusion process, at which the spikes are distributed to other nodes at a rate that is proportional to the connection strengths. Each increment of *τ* thus indicates an extra path between any two given regions (nodes), via *τ* − 1 intermediate nodes (e.g., a third-order connection between two nodes means they are connected via two other nodes; Figure 1B). In a continuous time interpretation of this process, we can equate connectivity with the number of spikes accumulated as time progresses. This interpretation is effectively a constrained diffusion process, where the diffusion coefficients are determined by the GL and, ultimately, every node is connected to every other node through multiple paths. This equilibrium distribution is the principle eigenmode of the matrix exponential of the GL (Figure 2H), revealing which nodes are more strongly involved in the diffusion or propagation process.

Diffusion simulation approaches capture the propensity of information or particle distribution along all possible paths in a network, and thus approximate network communicability (Estrada & Hatano, 2008; Crofts & Higham, 2009). Network communicability has already been used to characterise brain networks in normalcy and pathology, and in different species (Crofts & Higham, 2009; Andreotti et al., 2014; Grayson et al., 2016; Shine et al., 2018). In addition to routing efficiency representing shortest paths and thus easy and speedy communication between network nodes, taking into account recurrent neuronal message passing over multiple paths may afford more optimal approximations of brain dynamics (Bullmore & Sporns, 2012; Avena-Koenigsberger et al., 2017). Using the matrix exponential of the GL allows a path length-based correction of network communicability (Estrada & Hatano, 2008; Crofts & Higham, 2009). Previous applications of the GL in neuroscience have suggested it as a promising tool for modelling neurotransmitter diffusion in the synaptic junction (Barreda & Zhou, 2011), simulating the spread of neurodegeneration (Raj et al., 2012) and comparing resting-state functional with structural connectivity (Abdelnour et al., 2014, 2018).

The present study asks whether measures of simulated symmetric and asymmetric anatomical network diffusion may usefully inform the effective connectivity that underwrites causal and asymmetric interactions among distributed neuronal populations. We demonstrate the approach in the context of fMRI responses of a brain network to emotional body language, using probabilistic tractography on high angular resolution diffusion imaging (HARDI) data from the same cohort of normal individuals.

## MATERIALS AND METHODS

### Participants

We used fMRI and HARDI data from 17 right-handed, male normal subjects (mean age 27.9 years) from a study on emotional body language processing. The cohort overlapped with that analysed in previous research (Sokolov et al., 2012, 2014, 2018, 2019). The study was approved by the Ethics Committee of the University of Tübingen Medical School, Germany. Participants provided informed written consent and were financially compensated.

### fMRI and HARDI Data Recording and Preprocessing

A 3T scanner (TimTrio, Siemens Medical Solutions, Erlangen, Germany; 12 channel head coil) was used for recording of three-dimensional T1-weighted structural MRI (magnetisation-prepared rapid gradient echo, MPRAGE; 176 sagittal slices, TR = 2,300 ms, TE = 2.92 ms, TI = 1,100 ms, voxel size = 1 × 1 × 1 mm^3^), a field map for inhomogeneity correction, HARDI data (two sessions with 64 diffusion gradient directions per subject; b-value = 2,600 s/mm^2^, 54 axial slices, TR = 7,800 ms, TE = 108 ms, slice thickness = 2.5 mm, matrix size = 88 × 88, field of view = 216 mm) and functional echo-planar imaging (EPI; 171 volumes, 56 axial slices, TR = 4,000 ms, TE = 35 ms, in-plane resolution 2 × 2 mm^2^, slice thickness = 2 mm, 1 mm gap).

Participants viewed animations of an arm represented by bright dots placed on the head and main upper limb joints, facing to the right and knocking on an invisible door with different emotional content (happy, angry, neutral; Pollick et al., 2001; Sokolov et al., 2011). In an event-related design, the participants had to indicate which emotion was expressed by button press (button assignment counterbalanced between participants). Stimulus duration was 1,000 ms, and each stimulus category (emotion) was presented 30 times throughout the experiment. To optimise event-related response function estimation, we applied jittering of stimulus onset intervals (between 4,000 and 8,000 ms in steps of 500 ms) and stimulus order pseudo-randomisation.

Preprocessing of fMRI data was performed using Statistical Parametric Mapping software (SPM12, Wellcome Centre for Human Neuroimaging, Institute of Neurology, UCL, http://www.fil.ion.ucl.ac.uk/spm) and included slice timing correction, realignment, unwarping, image co-registration, segmentation-based normalisation, and smoothing. HARDI data preprocessing with the FMRIB’s Diffusion Toolbox within the FMRIB Software Library (FSL5, Oxford Centre for Functional MRI of the Brain, UK, http://www.fmrib.ox.ac.uk/fsl) consisted of brain extraction (Smith, 2002), motion and eddy current correction, followed by co-registration with the anatomical reference image and normalisation to Montreal Neurological Institute (MNI) space using the FMRIB Linear Image Registration Tool (FLIRT; Jenkinson et al., 2002). Gradient directions were adjusted according to the FLIRT parameters.

### fMRI Analysis and DCM Specification

Analysis of fMRI data was conducted by first specifying a general linear model (GLM). Trials with correctly classified emotional expression of point-light knocking (happy, angry, neutral) were assigned distinct regressors, and regressors of no interest were modelled for trials with incorrect classification (e.g., neutral stimulus classified as happy), trials with missing responses, six head motion parameters, and time series from WM and cerebrospinal fluid. The regressors were convolved with the haemodynamic response function. High-pass filtering was performed (cutoff 1/256 Hz), and serial autocorrelations were accounted for by a first-order autoregressive process (coefficient of 0.2) plus white noise model. The GLM was applied to individual preprocessed EPI data, and the contrasts happy versus neutral, angry versus neutral, and neutral versus emotional knocking were specified. Individual contrast images were submitted to second-level random effects analyses, and regional activations (at a *p* < 0.05 family-wise error corrected voxel-wise threshold for multiple comparisons) were identified using the automated anatomical labelling in SPM (Tzourio-Mazoyer et al., 2002) and the NeuroSynth.org database (http://neurosynth.org; Yarkoni et al., 2011).

A one-state, bilinear, and deterministic DCM with mean-centred inputs and reciprocal extrinsic connections between all nodes (full model) was created for each subject. This DCM included seven regions showing differential activation with respect to correctly classified emotional expressions of point-light knocking and three regions for activation versus baseline (Table 1). For each region, time series were extracted as the first eigenvariate of all activated voxels within a sphere with a radius of 8 mm, centred on each individual maximum (*p* < 0.05, uncorrected). The individual maxima were found within 7 mm of the group activation coordinate in every subject. The time series extraction was adjusted to remove effects that were not related to the task such as motion. According to previous data on the architecture of the brain network for body motion processing (Sokolov et al., 2018), driving input was specified on the left middle temporal cortex, right fusiform gyrus, and right superior temporal sulcus. Modulating input of different emotional content was expressed in the DCM B-matrix (see Supporting Information Methods) by modelling the influence of the corresponding regressors for happy, neutral, and angry stimuli on all extrinsic connections in the network, as well as on intrinsic coupling within the seven nodes showing differential activation depending on emotional content (Table 1).

**Table 1.** The regions forming the analysed network.

Anatomical label	MNI Coordinates			z-value	Cluster size
	X	Y	Z		
**Happy vs. neutral**					
R superior temporal sulcus (STS)	50	−38	8	5.82	186
R caudate nucleus (CAU)	10	18	4	5.46	120

**Angry vs. neutral**					
L midcingulate cortex (MCC)	−6	−6	−48	5.21	192
L anterior cingulate cortex (ACC)	−8	50	18	5.08	168
L insula (INS)	−28	14	−16	4.87	134

**Neutral vs. emotional**					
Cerebellar vermis, lobule IX (CRB)	0	−46	−48	6.02	206
R amygdala (AMY)	26	−4	−26	5.93	182

**Active (stimulation vs. baseline)**					
L middle temporal cortex (MTC)	−404	−784	−48	5.90	362
R fusiform gyrus (FFG)	18	−36	12	5.78	238
R retrosplenial cortex (RSP)	−6	−54	4	5.72	267

*Note*. Seven regions with differential activation to emotional expressions of point-light knocking and three regions showing activation not modulated by emotional content (at a *p* < 0.05 family-wise error corrected voxel-wise threshold for multiple comparisons) were included in the analysis. Regional labels are provided along with coordinates in MNI space, corresponding z-values, and cluster sizes.

### Direct Structural Adjacency Matrix

Individual preprocessed and normalised HARDI data were submitted to Bayesian Estimation of Diffusion Parameters Obtained using Sampling Techniques with modelling of Crossing Fibres (BEDPOSTX; Behrens et al., 2007) in FSL to obtain diffusion parameters for each voxel. Probabilistic tractography with crossing fibres (PROBTRACKX; step length = 0.5 mm, number of steps = 2,000, number of pathways = 5,000, curvature threshold = 0.2, modified Euler integration; Behrens et al., 2007) was performed for each DCM node as a seed, and the other DCM nodes as classification targets. The fibre pathway outputs were visually controlled for plausibility. Structural connection strength between a seed region *i* and a classification target region *j* was obtained by averaging the number of streamlines connecting every voxel in *i* to one voxel of *j*, across both directions of tractography. Further averaging across all subjects provided a symmetric group structural adjacency matrix (Figure 2A). We eschewed thresholding and considered weighted adjacency matrices. Each element of the group adjacency matrix *Z* was normalised to represent direct structural connection strength or probability φ, relative to the greatest connection strength within the matrix. The between-region elements of matrix *Z* were used to inform models of effective connectivity by direct structural connectivity, and for GL-based simulation of network diffusion to obtain measures of indirect structural connectivity.

### Graph Laplacian

We used the GL matrix *L* to construct a connectivity operator simulating diffusion of a conserved quantity (heat, spikes) along direct and indirect pathways between the network nodes of the structural adjacency matrix *Z*. As per definition, each column of the GL matrix *L* expressing probabilities φ of extrinsic structural connections has to sum to zero, which consequently applies to any linear mixture of the columns of *L*.

To achieve this, we set the leading diagonal elements of *L* to the negative sum of the corresponding column of *Z* (Supporting Information Figure S1):

L=Z−D (1)

where *D* is the degree matrix *D*_*i*,*i*_ = $\sum_{i=1}^{n}$ *Z*_*i*,*j*_.

We obtained our connectivity operator by calculating the matrix exponential Γ of *L*:

$$\Gamma =\exp(L)=\sum_{k=1}^{\infty }\frac{1}{k!}L^{k}$$ (2)

Subsequent exponentiation of Γ simulated distribution of particles (spikes) and thus yielded indirect, high-order structural connectivity *ψ* after every time step *τ*:

$$\Psi (\tau )=\Gamma^{\tau }$$ (3)

Γ^0^ corresponds to the identity matrix, and the first order of the matrix exponential of the GL Γ^1^ represents the distribution of particles ψ(1) after a first time point of diffusion *τ* = 1 (Figure 2B). With every increase of *τ* (order of Γ^*τ*^), another time step is calculated and indirect connections *ψ*(*τ*)_*i*,*j*_ between nodes *i* and *j* become apparent or reinforced. Inherently, this simulated distribution of a conserved quantity constrained by the structural connectivity saturates after a certain, unknown number of diffusion steps. We tested our hypothesis that this state of equilibrium or saturation would provide the most informative priors on effective connectivity by comparing DCMs with indirect structural connectivity priors at different time steps (orders) *τ* of the GL diffusion process.

Furthermore, we hypothesised that simulated network diffusion on asymmetric structural adjacency matrices may introduce more plausible constraints on asymmetric effective brain connectivity. Accordingly, we introduced three variants of the adjacency matrix *Z* as the basis for the diffusion process (Supporting Information Figure S1): (1) the symmetric adjacency matrix *Z* normalised to its maximum, (2) asymmetric *Z*′ normalised along the rows, and (3) asymmetric *Z*′ normalised along the columns in the following way:

$$Z^{\prime }=\frac{Z}{W}$$ (4)

with normalisation of *Z* to its out-degree (setting the sum ofweights in each column to unity) when the diagonal degree matrix is *W*_*i*,*i*_ = $\sum_{i=1}^{n}$ *Z*_*i*,*j*_ and normalisation of *Z* to its in-degree (sum of weights in each row set to unity) for *W*_*i*,*i*_ = $\sum_{j=1}^{n}$ *Z*_*i*,*j*_. When normalising to the out-degree, we assume that each node has a fixed capacity to influence other nodes, and that the relative influence is proportional to the efferent diffusion process along structural pathways. Conversely, normalisation to the in-degree means that each target node has a fixed capacity to be influenced by other nodes and its relative sensitivity is determined by the probability of receiving input during the diffusion process. In what follows, we describe the evaluation of which indirect structural connectivity *ψ*(*τ*) within each of the three plausible normalisation schemes underlying the diffusion operator Γ^*τ*^ afforded the best constraints on effective connectivity.

### Integration of Structural Connectivity with Dynamic Causal Modelling

After defining prior beliefs about the effective connectivity parameters, the estimation of DCMs affords posterior estimates of the parameters as well as the evidence for the respective model (Friston et al., 2003, Supporting Information Methods). The priors for extrinsic (off-diagonal; between-region) connections in dynamic causal modelling form a multivariate normal distribution, defined by a vector of expectations and a prior covariance matrix Σ_*y*_. By default, the prior expectation is zero and the variance is equal across all extrinsic connections. The greater the prior variance, the further the connectivity parameters can deviate from their prior expectation of zero.

The priors also contribute to the calculation of the model evidence—the quantity used to compare DCMs—which is the trade-off between model accuracy and complexity. In this context, complexity is defined as the discrepancy between prior assumptions and posterior estimates, where greater complexity decreases model evidence. Optimising the priors according to measures of structural connectivity may therefore increase model evidence, through a reduction of model complexity (Stephan et al., 2009; Sokolov et al., 2019).

However, the precise relationship between structure and function is unknown and likely varies for different networks. Previous research provided support for the intuition that the strength of direct structural connections relates to the prior effective connectivity in a positive monotonic fashion (Stephan et al., 2009; Sokolov et al., 2018, 2019). This means that for lower structural connection strengths, the prior variance shrinks to a small value, precluding strong effective connectivity. Conversely, for greater structural connection strengths, our prior belief that the effective connectivity is close to zero can be relaxed by increasing the prior variance.

Based on this rationale, to assess the utility of direct structural connectivity as priors for effective connectivity, we used our previously developed structurally informed parametric empirical Bayes (si-PEB) approach (Sokolov et al., 2019) to obtain the reduced prior covariance Σ_*y red*_ from the probability *φ* for direct structural connectivity encoded in the symmetric structural adjacency matrix *Z* normalised to its maximum:

$$\Sigma_{y\;red}=\frac{\Sigma_{y\;max}}{1+\exp(\alpha -\delta *\phi )}$$ (5)

where maximum prior covariance is determined by the hyperparameter Σ_*y max*_ (range from 0.0625 to 0.25 in four equal steps), the sigmoid slope by *δ* (range from 0 to 16 in eight equal steps) and sigmoid shift by *α* (range −2 to 2 in eight equal steps). This hyperparameter space yielded 405 different mappings or models per network.

For indirect structural connectivity, across the three normalisation schemes (see Equation 4), by assuming a simple linear positive relationship, we mapped the logarithm of indirect structural connection probability *ψ*(*τ*) afforded by the *τ*-th order of the diffusion operator Γ^*τ*^ to the prior covariance as follows:

$$\Sigma_{y\;red}=\Sigma_{y\;min}+\delta *(log(\psi (\tau )-e^{-b})+b)$$ (6)

Here, similar to Equation 5, the three-dimensional hyperparameter model space is spanned by the hyperparameters *τ* (range 1–64 in seven equal steps for both networks), Σ_*y min*_ (representing the default prior covariance, range 0.0156–0.0625 in seven equal steps for both networks), and *δ* (sigmoid slope; range 0–0.25 in seven equal steps). The space thus contains 512 different models per normalisation scheme, or 1,536 models in total. The use of log-transformed structural probabilities *ψ*(*τ*) is motivated easily by noting that most structural connections have a log normal distribution, and indeed have an exponential dependency upon distance (Markov et al., 2013). Here, *b* is a small number that ensures the prior variance Σ_*y min*_ over effective connectivity is lower bounded; in the absence of structural connectivity: Σ_*y red*_ = Σ_*y min*_.

Crucially, both model spaces (for direct and indirect structural connectivity) include flat mappings (i.e., *δ* = 0 ), where structural constraints do not matter and Σ_*y red*_ is the same across all extrinsic connections, thus representing an intrinsic control (null hypothesis) for the assumption that structural constraints usefully shape effective connectivity.

### Model Estimation and Evaluation

As we analysed second-level measures of structural connectivity, we used parametric empirical Bayes (PEB) (Friston et al., 2015; Supporting Information Methods) to make inferences on effective connectivity at the group level. PEB is a hierarchical model, in which the average group connectivity acts as an empirical prior on individual connectivity (Friston et al., 2016). PEB estimation thus represents an iterative process between individual and group effective connectivity. Therefore, PEB properly partitions within- and between-subject random effects (Zeidman et al., 2019) and is robust to local minima problems (Friston et al., 2016). The individual DCMs were estimated using the PEB scheme with a prior variance of 0.5 for all extrinsic connections. Subsequently, the prior PEB variance of each extrinsic connection was adapted (reduced) using Equations 5 and 6 for measures of direct and indirect structural connection strengths, respectively.

The search for the models with the greatest evidence was afforded by Bayesian model reduction (BMR) (Friston et al., 2016; Supporting Information Methods). This recently introduced statistical device enables analytical evaluation of large model spaces in a matter of seconds, based on the estimation of a single, so-called “full” model. In contrast, the use of conventional dynamic causal modelling would have required separate estimation of each of the 1,941 alternative models (estimated processing duration: 1,300 days). By comparing the log evidences of the different models, we assessed whether effective connectivity was better explained by (1) indirect as opposed to direct measures of structural connectivity, (2) a particular order of the connectivity operator Γ^*τ*^, and (3) a particular normalisation scheme of the structural adjacency matrix underlying Γ. Very strong evidence that one model provides a better account for the observed data than another is concluded from a relative log-model evidence of three (Penny, 2012), corresponding to a posterior probability of 95% or above.

## RESULTS

### Anatomical Network Diffusion Outperformed Direct Pathways in Sculpting Effective Connectivity

We assessed the value of simulated indirect (high-order) anatomical connectivity afforded by network diffusion under the GL for sculpting effective connectivity, relative to models informed by direct structural connectivity and to DCMs without anatomical information.

For indirect structural connectivity, we used a sigmoid function (Equation 6) with the hyperparameters *δ* (slope; range 0–0.25 in seven equal steps) and Σ_*y min*_ (lower boundary on prior variance; range 0.0156–0.0625 in seven equal steps) to map the log-transformed group connectivity values provided at eight different, equally distributed orders *τ* of the diffusion process (from 1 to 64) onto prior variance of second-level effective connectivity.

**Figure 3.**
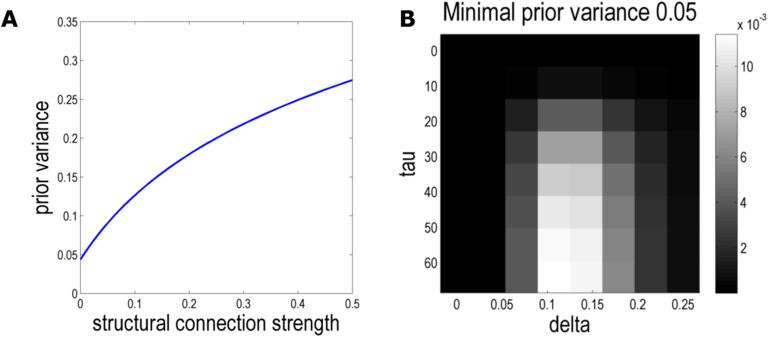
Mapping indirect anatomical to effective connectivity priors. (A) The mapping from indirect structural connectivity to prior second-level variance for the optimal combination of hyperparameters *τ* = 64, *δ* = 0.15, and Σ_*y min*_ = 0.05. (B) BMR affords the posterior probability for each model informed by indirect structural connectivity relative to a full, uninformed model with a uniform prior variance of 0.5 for all extrinsic (between-region) connections. Higher luminosity represents greater posterior probability. At every time step (order *τ*) of the graph Laplacian, Equation 6 is applied to map the resulting structural connectivity to prior variance on second-level effective connectivity, defined by the sensitivity hyperparameter *δ*. The posterior probabilities computed from the log model evidences are shown for the optimal prior second-level variance (Σ_*y min*_ = 0.05). The important aspect of this distribution is that the highest posterior probabilities (brightest grids) are observed for a sensitivity hyperparameter *δ* substantially greater than zero (that would mean structural connectivity does not provide useful constraints) and for higher orders *τ* of indirect structural connectivity.

A grid search over the 1,536 candidate models resulting from the diffusion process under three normalisation schemes (symmetric, weighted to the out-degree, weighted to the in-degree) using BMR (overall computation time 5.98 seconds) indicated the best constraints on effective connectivity were provided by the largest order (*τ* = 64) of a GL normalised to the in-degree. The structure-function mapping at this order was governed by the hyperparameters *δ* = 0.15 and Σ_*y min*_ = 0.05 (Figure 3).

This model clearly outperformed models informed by direct anatomical connectivity (log-evidence difference 33.13 in favour of indirect structural connectivity) and those without anatomical information (log-evidence difference 35.3 in favour of indirect structural connectivity). Very strong evidence that one model provides a better account for the observed data than another is concluded for a log-evidence difference of three or above (Penny, 2012).

### The Graph Laplacian Principle Eigenmode Aligned with Effective Connectivity

As shown in Figure 3B, the evidence for DCMs of effective connectivity informed by indirect structural connectivity priors increased with progression of the particle diffusion process simulated by the GL, saturating at orders above *τ* = 50, corresponding to the principle eigenmode of the GL. This suggests the structural connectivity that matters for dynamical coupling and effective connectivity is best conceived in terms of reciprocal message passing over long (polysynaptic) paths, or periods of time.

### Normalisation Schemes: Afference Matters

**Figure 4.**
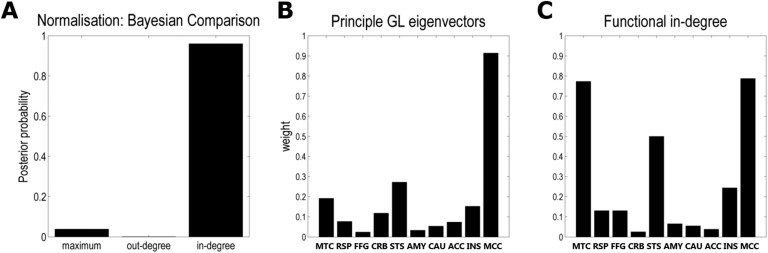
The role of asymmetry. (A) The bars represent the posterior probabilities of the most probable model in each of the three different normalisation schemes (to the maximum, to the out-degree and to the in-degree). This analysis indicated very strong evidence in favour of normalisation to the in-degree (posterior probability 96%). (B) The bars illustrate the first GL eigenvectors for each node (equivalent to the horizontal bands in the equilibrium state, Figure 2H). They showed that the midcingular cortex (MCC), superior temporal sulcus (STS), middle temporal cortex (MTC), and insula (INS) received most particles during the diffusion process (in descending order). (C) Crucially, these four network nodes were also those with the highest functional input (in-degree) based on effective connectivity. Here, the functional input (bars) for each node was defined as equalling the sum of squared weights over the respective rows of the effective connectivity matrix. Taken together, this indicates that effective connectivity is best constrained by the input sensitivity of the network nodes in the diffusion process simulated by the GL. Please see Table 1 for all abbreviated region labels.

Bayesian model comparison (Penny, 2012) across the three normalisation schemes provided consistently very strong evidence in favour of normalisation to the in-degree (log-evidence difference between this and the next probable normalisation scheme: 3.06; Figure 4). This result implied that effective connectivity is best predicted by the relative sensitivity of nodes to incoming information or afference and confirmed our hypothesis that introduction of asymmetry in the diffusion process may offer a more plausible characterisation of asymmetric brain dynamics.

When examining how the principle eigenmode of the GL normalised to the in-degree related to the functional afference of each node, one can see that the four nodes receiving the greatest input during the GL diffusion process (midcingular cortex (MCC), superior temporal sulcus (STS), middle temporal cortex (MTC) and insula (INS)) were also those with the highest functional in-degree based on effective connectivity (Figure 4). This further speaks to the utility and construct validity of the GL approach to inform effective by indirect structural connectivity.

### Permutation Testing

**Figure 5.**
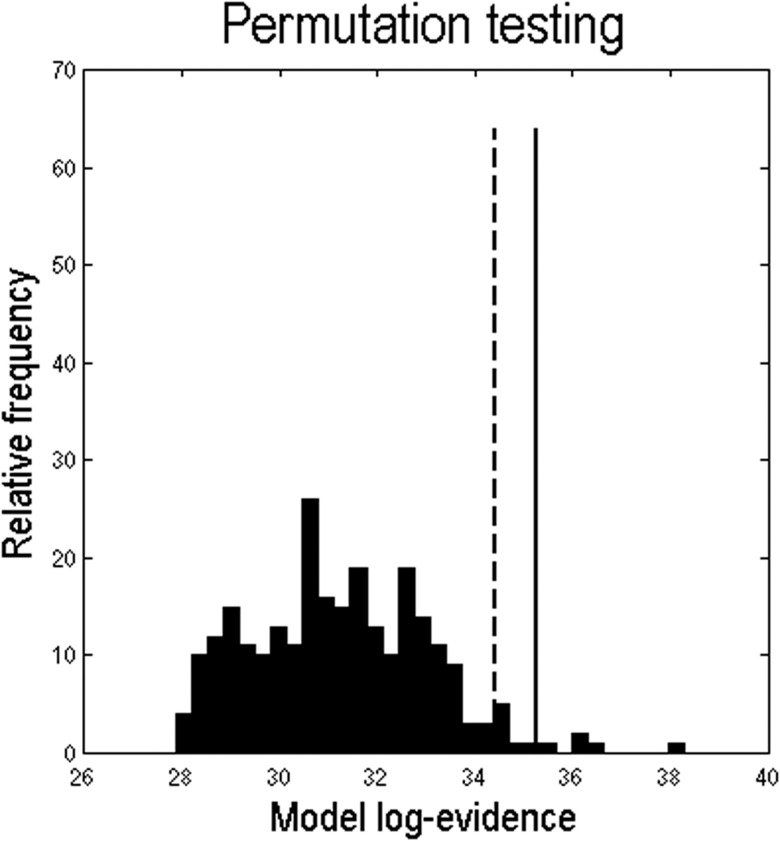
Permutation testing with random structural adjacency matrices. In order to assess how often the graph Laplacian diffusion process over a random structural adjacency matrix would produce a log evidence greater than that afforded by the true structural adjacency matrix, 256 matrices were formed by randomly permuting the network nodes, thereby preserving the symmetry and distribution of edge weights. The histogram shows the distribution of maximum log evidences over these different models (under in-degree normalisation). The dashed line corresponds to a threshold of *p* = 0.05 (a significant result should be located to the right of this line), while the solid line is the observed log evidence for the best empirical model. The result indicates the improvement in model evidence afforded by the true tractography matrix is significant (*p* = 0.01) in a classical sense, with respect to a null distribution based on permutation testing.

Random permutations (*n* = 256) of the network nodes in the adjacency matrix (thereby preserving the distribution of edge weights) were used to assess how often a random structural adjacency matrix would afford a greater log-model evidence than reported above (after optimising the normalisation, order, and sigmoid hyperparameters). This permutation testing suggested the improvement in evidence afforded by applying the GL to the actual structural adjacency matrix was significant in a classical sense (*p* = 0.01), with respect to a null distribution of largest log-model evidences (Figure 5).

## DISCUSSION

This study makes several contributions to the understanding of structure-function relationships in the brain. Based on previous research (Kale et al., 2018; Avena-Koenigsberger et al., 2019; Seguin et al., 2019), we hypothesised that asymmetric polysynaptic anatomical connectivity would better predict the directed causal dynamics between neuronal populations (effective connectivity) than conventional (i.e., symmetric and monosynaptic) information on WM pathways. The introduction of the GL allowed us to parameterise a diffusion process on the structural graph, providing symmetrically and asymmetrically weighted adjacency matrices of increasing order. Of note, other methods for modelling network communication based on structural connectomes can inherently inform on asymmetry. Such approaches include navigation (Seguin et al., 2018), search information (Goni et al., 2014), linear transmission models (Misic et al., 2015), and diffusion efficiency (Goni et al., 2013). The novel approach presented here enables hypotheses to be tested about the mapping from indirect anatomical connectivity to effective connectivity via a (variational) Bayesian framework. Using a dataset with fMRI and HARDI measures, we found that high-order structural connectivity simulated using the GL greatly improved the evidence for DCMs of effective connectivity, compared to DCMs informed by direct structural connectivity and anatomically uninformed models. Most importantly, input sensitivity during the diffusion process best predicted effective connectivity.

We introduced a computationally efficient approach to map indirect anatomical to effective connectivity, using hierarchical PEB models and BMR for DCMs (Friston et al., 2016; Zeidman et al., 2019). This procedure is designed to account inherently for possible variations in network architecture, normalisation scheme, value of anatomical information and mapping between structural and effective connectivity, for any given study and context. By definition, effective connectivity is determined by the context, such as the specific experimental task or cognitive set (Friston et al., 2003). For this reason, we would not expect a universally optimal set of priors on effective connectivity that could explain cognition per se. We sought to provide an efficient method for finding the best effective connectivity priors for any specific context, informed by indirect anatomical connectivity. An interesting future question will be whether the utility of the GL approach to assess indirect structural connectivity generalises to models of effective connectivity for resting-state data. The value of understanding asymmetric coupling between functionally related regions at rest, through combination of neuronal and observational models such as those used by dynamic causal modelling, has become increasingly recognised (Friston et al., 2014; Razi et al., 2015, 2017). Recent work has already linked asymmetries in indirect anatomical connectivity to resting-state effective connectivity (Seguin et al., 2019).

Using high-quality dMRI along with task-related and resting-state fMRI datasets such as from the Human Connectome Project (Van Essen et al., 2013) may contribute to further test and refine the outlined approach and conclusions, complementing previous research on these datasets (Seguin et al., 2019). Furthermore, it will be of interest to perform large-scale analyses integrating the structural connectome with whole-brain effective connectivity using the recently introduced regressive dynamic causal modelling (Frassle et al., 2018). Such approaches may also help to inform generative models of how WM pathways give rise to brain dynamics (Robinson, 2012; Deco et al., 2013; Sanz Leon et al., 2013; Melozzi et al., 2017; Messe et al., 2018), and to better understand how network dynamics may shape cognitive function and behaviour (Aertsen et al., 1994; Gerraty et al., 2018; Sokolov et al., 2018). Relating the diffusion properties of brain networks to their functions in extension of previous approaches based on direct anatomical connectivity (Deco et al., 2012; Senden et al., 2012; Hermundstad et al., 2014) may further improve our conceptualisation of distributed information processing in the brain.

Endowing the GL diffusion process with asymmetry clearly outperformed a symmetric diffusion process in sculpting effective connectivity, suggesting that ensemble dynamics in the brain may be shaped by the sensitivity of regions to their distributed input. These findings agree with and extend recent work on resting-state functional connectivity in macaques and humans showing that synchrony between nodes does not only depend on their direct or second-order connectivity, but also the similarity of afferents they receive from the entire network and other adjacent network characteristics (Adachi et al., 2012; Goni et al., 2014; Bettinardi et al., 2017). Consequently, densely connected regions may not necessarily be in a best position to influence or to be influenced by other regions (Avena-Koenigsberger et al., 2017). This follows from the fact that being locked into dense subgraphs may preclude a more widespread sensitivity to distributed dynamics (Pillai & Jirsa, 2017). Clarifying whether and why some networks may be better characterised in terms of their nodes’ sensitivity to inputs as opposed to their capacity to influence other nodes is a promising avenue for future research on normal and altered brain network function that can be pursued formally by the procedure described here.

The results presented here agree with and extend previous research employing network communication models (Avena-Koenigsberger et al., 2019; Seguin et al., 2019). These studies inferred the ease of sending and receiving information from undirected structural connectomes. The differences between send and receive efficiencies were mapped onto functional brain topography and the outcomes of separate resting-state effective connectivity analyses. For instance, anatomical connectivity-based classification suggested that unimodal areas such as the primary visual cortex or sensorimotor cortices were predominantly sending information, whereas multimodal regions were mainly receivers (Seguin et al., 2019). The diffusion efficiency approach, described as the (inverse) mean first passage time of a Markov chain process (Goni et al., 2013; Seguin et al., 2019), is similar to in-degree normalisation. In contrast to the present work, diffusion efficiency is derived using a matrix of transition probabilities. Nonetheless, measuring diffusion efficiency in a structural adjacency matrix yielded similar results, revealing regional variability in input sensitivity and a rather uniform capacity to influence other regions (Seguin et al., 2019). Future investigations are needed to fully explore the implications of the various measures to model diffusion processes.

The other important finding was that higher orders and the principle eigenmode of the GL afforded better priors on effective connectivity than lower GL orders. This indicated that WM connections and distributed neural dynamics give rise to brain communication through recurrent neuronal message passing over multiple paths. GL eigenmodes and eigenvalues are closely related to network communicability, representing the ease of information transmission along all possible paths in a network (Estrada & Hatano, 2008; Crofts & Higham, 2009; Andreotti et al., 2014; Grayson et al., 2016; Shine et al., 2018). GL eigenmodes of structural adjacency matrices exhibit a high degree of similarity between healthy subjects, as well as consistent and meaningful alterations in developmental and virtual agenesis of the corpus callosum (Wang et al., 2017). Laplacian eigenvalue spectra have been used for cross-species comparison of anatomical networks and revealed specific characteristics of neural networks as opposed to other network classes (de Lange et al., 2014). Furthermore, the anatomical graph energy (connectedness measure representing the sum of all absolute GL eigenvalues) has been shown to be significantly lower in patients with Alzheimer’s disease than in controls (Daianu et al., 2015). A greater number of apolipoprotein E4 gene copies predicted this reduction in graph energy. The use and interpretation of metrics such as eigenmodes and eigenvalues afforded by diffusion processes simulated by the exponentiation of a GL matrix could lead towards consideration of more global network characteristics beyond the conventionally assessed single hub or subgraph properties.

In clinical research, truly integrative computational, graph theoretic, or even simple correlative analyses of multimodal connectivity remain rather sparse. However, the assessment and comparison of network communicability using the GL may be of potential relevance to clinical neuroscience. Implementation of the GL in patients to assess how local and global changes in anatomical connectivity affect functional dynamics may shed new light on pathophysiology. Other comparative network measures afforded by the GL are topological similarity, persistent homology and graph diffusion distance (Hammond et al., 2013a; Bettinardi et al., 2017; Liang & Wang, 2017). Furthermore, simulation of network diffusion by means of the GL has been used to predict neuronal spreading and resulting brain atrophy patterns in Alzheimer’s and frontotemporal dementia (Raj et al., 2012) and to infer sources of disease propagation in mild cognitive impairment and Alzheimer’s dementia (Hu et al., 2016). Ultimately, the global measures afforded by the GL may be used towards assessment of the relationships between connectivity and behaviour at the network level (Sokolov et al., 2018). As efficiency and ease-of-use are of primary significance in everyday clinical practice, this relatively straightforward and rapid approach could potentially afford useful network biomarkers in neurological and psychiatric disorders.

Dynamic causal modelling for fMRI has contributed to establishing or refining various neuroanatomical and neurobiological concepts and hypotheses in functional realms such as reading, mental imagery, memory retrieval, or body language reading (Chow et al., 2008; Sokolov et al., 2012; Dijkstra et al., 2017; Ren et al., 2018; Sokolov et al., 2018). Inclusion of electrophysiological data from EEG, MEG, or intracranial recording (David et al., 2013; Almashaikhi et al., 2014; Proix et al., 2017), which enable more detailed biophysical modelling due to their high temporal resolution, may offer additional insight. Indeed, structure-function relationships appear to depend on different timescales of functional dynamics. Changes over periods of several minutes largely reflect underlying anatomical connectivity, whereas dynamics lasting a few seconds do so to a lesser degree (Honey et al., 2007; Shen et al., 2015; Cabral et al., 2017). Interestingly, resting-state functional connectivity data derived from simultaneous EEG and fMRI outperforms fMRI data alone in modelling structural connectivity, when comparing these predictions to actual dMRI measures (Wirsich et al., 2017). Network models based on anatomical connectivity and driven by EEG source activity accurately predict individual fMRI resting-state patterns and reproduce neurophysiological phenomena and mechanisms observed with different imaging modalities (Schirner et al., 2018). GL approaches to structural connectivity have also been used to improve EEG source localisation (Hammond et al., 2013b). Another potentially exciting development could be the computational modelling of sparse impulse stimulations as input in the GL diffusion process, similar to that implemented for modelling of disease propagation sources (Hu et al., 2016). This could further approximate the neuronal model within dynamic causal modelling (Friston et al., 2003) and information flow in the brain.

In summary, we have studied symmetric and asymmetric diffusion processes based on anatomical connectivity to optimise prior constraints on models of directed effective connectivity. Bayesian model comparison indicated the best effective connectivity priors are provided by indirect, high-order structural connectivity determined by the regional sensitivity to inputs that would be seen under equilibrium states of particle diffusion within an anatomical network. This may speak to a reappraisal of how we characterise the anatomical connectome, when trying to understand asymmetric functional dynamics arising from structure of the sort measured in neuroimaging.

## ACKNOWLEDGMENTS

The authors wish to thank Richard Frackowiak, Patric Hagmann, Alexander Sokolov, and Klaas-Enno Stephan for valuable discussion. Technical support was provided by Ric Davis, Jürgen Dax, Chris Freemantle, Bernd Kardatzki, Rachael Maddock and Liam Reilly, and administrative assistance by Marcia Bennett, David Blundred, Kamlyn Ramkissoon, Tracy Skinner, and Daniela Warr.

## SUPPORTING INFORMATION

Supporting Information for this article is available at https://doi.org/10.1162/netn_a_00150.

## AUTHOR CONTRIBUTIONS

Arseny A. Sokolov: Conceptualization; Formal analysis; Funding acquisition; Investigation; Methodology; Writing - Original Draft. Peter Zeidman: Methodology; Supervision; Writing - Review & Editing. Adeel Razi: Conceptualization; Methodology; Writing - Review & Editing. Michael Erb: Methodology; Supervision. Philippe Ryvlin: Supervision; Writing - Review & Editing. Marina A. Pavlova: Conceptualization; Funding acquisition; Methodology; Resources; Supervision; Writing - Review & Editing. Karl J. Friston: Conceptualization; Formal analysis; Funding acquisition; Methodology; Resources; Supervision; Writing - Review & Editing.

## FUNDING INFORMATION

Arseny A. Sokolov, Baasch-Medicus Foundation. Arseny A. Sokolov, Fondation Leenaards (http://dx.doi.org/10.13039/501100006387). Arseny A. Sokolov, Schweizerische Neurologische Gesellschaft (http://dx.doi.org/10.13039/100010766). Arseny A. Sokolov, Helmut Horten Foundation. Arseny A. Sokolov, Synapsis Foundation Alzheimer Research Switzerland, Award ID: 2019-CDA03. Marina A. Pavlova, Reinhold Beitlich Stiftung (http://dx.doi.org/10.13039/501100003541). Marina A. Pavlova, BBBank Foundation. Marina A. Pavlova, Deutsche Forschungsgemeinschaft (http://dx.doi.org/10.13039/501100001659), Award ID: DFG PA 847/22-1. Karl J. Friston, Wellcome Trust (http://dx.doi.org/10.13039/100004440), Award ID: 088130/Z/09/Z.

## TECHNICAL TERMS

Effective connectivity: A measure of the directed (causal) influence of one neural system over another using a model of neuronal interactions.
Dynamic causal modelling: A Bayesian framework which is used to infer causal interactions between coupled or distributed neuronal systems (effective connectivity).
High-order structural connectivity: Structurally unconnected regions communicate polysynaptically to engender indirect connectivity over multiple hops.
Graph Laplacian: A matrix representation of a graph that combines node adjacency and node degree in mathematical formulation and belongs to spectral graph theory.
Spectral graph theory: A study of the relationship between a graph and the eigenvalues and eigenvectors of its Laplacian matrix.
Adjacency matrix: Square matrix representation of a graph which is either binary (presence or absence of connections) or weighted (showing strength of connections).
Eigenmode: A stable state (i.e., mode) of a dynamic system in which all parts of the system oscillate at the same frequency.
Network communicability: A graph-theoretic measure of the ease of information propagation in a network of interconnected regions.
Routing efficiency: A measure of communication efficiency, representing the average inverse shortest path length between all pairs of nodes in a complex network.
Network diffusion: A process simulating the propagation of heat (or information) within a network.
Probabilistic tractography: Estimation of white matter pathway trajectories based on diffusion MRI data.
Model evidence: The model evidence, or marginal likelihood, represents the probability of observing the measured data under a certain model and is used for Bayesian model comparisons.

## References

[bib1] Abdelnour, F., Dayan, M., Devinsky, O., Thesen, T., & Raj, A. (2018). Functional brain connectivity is predictable from anatomic networks Laplacian eigen-structure. Neuroimage, 172, 728–739. 10.1016/j.neuroimage.2018.02.016 29454104 PMC6170160

[bib2] Abdelnour, F., Voss, H. U., & Raj, A. (2014). Network diffusion accurately models the relationship between structural and functional brain connectivity networks. Neuroimage, 90, 335–347. 10.1016/j.neuroimage.2013.12.039 24384152 PMC3951650

[bib3] Adachi, Y., Osada, T., Sporns, O., Watanabe, T., Matsui, T., Miyamoto, K., & Miyashita, Y. (2012). Functional connectivity between anatomically unconnected areas is shaped by collective network-level effects in the macaque cortex. Cerebral Cortex, 22(7), 1586–1592. 10.1093/cercor/bhr234 21893683

[bib4] Aertsen, A., Erb, M., & Palm, G. (1994). Dynamics of functional coupling in the cerebral cortex: An attempt at a model-based interpretation. Physica D: Nonlinear Phenomena, 75(1–3), 103–128.

[bib5] Almashaikhi, T., Rheims, S., Jung, J., Ostrowsky-Coste, K., Montavont, A., De Bellescize, J., Arzimanoglou, A., Keo Kosal, P., Guenot, M., Bertrand, O., & Ryvlin, P. (2014). Functional connectivity of insular efferences. Human Brain Mapping, 35(10), 5279–5294. 10.1002/hbm.22549 24839121 PMC6869741

[bib6] Andreotti, J., Jann, K., Melie-Garcia, L., Giezendanner, S., Abela, E., Wiest, R., Dierks, T., & Federspiel, A. (2014). Validation of network communicability metrics for the analysis of brain structural networks. PLoS One, 9(12), e115503 10.1371/journal.pone.0115503 25549088 PMC4280193

[bib7] Avena-Koenigsberger, A., Misic, B., & Sporns, O. (2017). Communication dynamics in complex brain networks. Nature Reviews Neuroscience, 19(1), 17–33. 10.1038/nrn.2017.149 29238085

[bib8] Avena-Koenigsberger, A., Yan, X., Kolchinsky, A., van den Heuvel, M. P., Hagmann, P., & Sporns, O. (2019). A spectrum of routing strategies for brain networks. PLoS Computational Biology, 15(3), e1006833 10.1371/journal.pcbi.1006833 30849087 PMC6426276

[bib9] Barreda, J. L., & Zhou, H. X. (2011). A solvable model for the diffusion and reaction of neurotransmitters in a synaptic junction. BMC Biophysics, 4, 5 10.1186/2046-1682-4-5 21596000 PMC3093673

[bib10] Becker, C. O., Pequito, S., Pappas, G. J., Miller, M. B., Grafton, S. T., Bassett, D. S., & Preciado, V. M. (2016). Accurately predicting functional connectivity from diffusion imaging. In arXiv.p1512.02602v02603. 10.1038/s41598-017-18769-x PMC5780460 29362436

[bib11] Behrens, T. E., Berg, H. J., Jbabdi, S., Rushworth, M. F., & Woolrich, M. W. (2007). Probabilistic diffusion tractography with multiple fibre orientations: What can we gain?. Neuroimage, 34(1), 144–155. 10.1016/j.neuroimage.2006.09.018 17070705 PMC7116582

[bib12] Bettinardi, R. G., Deco, G., Karlaftis, V. M., Van Hartevelt, T. J., Fernandes, H. M., Kourtzi, Z., Kringelbach, M. L, & Zamora-Lopez, G. (2017). How structure sculpts function: Unveiling the contribution of anatomical connectivity to the brain’s spontaneous correlation structure. Chaos, 27(4), 047409 10.1063/1.4980099 28456160

[bib13] Biggs, N. L. (1993). Algebraic graph theory. Cambridge: Cambridge University Press.

[bib14] Bullmore, E., & Sporns, O. (2012). The economy of brain network organization. Nature Reviews Neuroscience, 13(5), 336–349. 10.1038/nrn3214 22498897

[bib15] Cabral, J., Kringelbach, M. L., & Deco, G. (2017). Functional connectivity dynamically evolves on multiple time-scales over a static structural connectome: Models and mechanisms. Neuroimage, 160, 84–96. 10.1016/j.neuroimage.2017.03.045 28343985

[bib16] Chow, H. M., Kaup, B., Raabe, M., & Greenlee, M. W. (2008). Evidence of fronto-temporal interactions for strategic inference processes during language comprehension. Neuroimage, 40(2), 940–954. https://doi.org/S1053-8119(07)01089-0 18201911 10.1016/j.neuroimage.2007.11.044

[bib17] Crofts, J. J., & Higham, D. J. (2009). A weighted communicability measure applied to complex brain networks. Journal of the Royal Society Interface, 6(33), 411–414. 10.1098/rsif.2008.0484 PMC2658663 19141429

[bib18] Daianu, M., Mezher, A., Jahanshad, N., Hibar, D. P., Nir, T. M., Jack, C. R., Jr., Weiner, M. W., Bernstein, M. A., & Thompson, P. M. (2015). Spectral graph theory and graph energy metrics show evidence for the Alzheimer’s disease disconnection syndrome in Apoe-4 risk gene carriers. Proceedings IEEE International Symposium on Biomedical Imaging, 2015, 458–461. 10.1109/ISBI.2015.7163910 26413205 PMC4578320

[bib19] David, O., Job, A. S., De Palma, L., Hoffmann, D., Minotti, L., & Kahane, P. (2013). Probabilistic functional tractography of the human cortex. Neuroimage, 80, 307–317. 10.1016/j.neuroimage.2013.05.075 23707583

[bib20] Deco, G., Ponce-Alvarez, A., Mantini, D., Romani, G. L., Hagmann, P., & Corbetta, M. (2013). Resting-state functional connectivity emerges from structurally and dynamically shaped slow linear fluctuations. Journal of Neuroscience, 33(27), 11239–11252. 10.1523/JNEUROSCI.1091-13.2013 23825427 PMC3718368

[bib21] Deco, G., Senden, M., & Jirsa, V. (2012). How anatomy shapes dynamics: A semi-analytical study of the brain at rest by a simple spin model. Frontiers in Computational Neuroscience, 6, 68 10.3389/fncom.2012.00068 23024632 PMC3447303

[bib22] de Lange, S. C., de Reus, M. A., & van den Heuvel, M. P. (2014). The Laplacian spectrum of neural networks. Frontiers in Computational Neuroscience, 7, 189 10.3389/fncom.2013.00189 24454286 PMC3888935

[bib23] Deligianni, F., Robinson, E., Beckmann, C. F., Sharp, D., Edwards, A. D., & Rueckert, D. (2011). Inference of functional connectivity from direct and indirect structural brain connections. In 2011 IEEE International Symposium on Biomedical Imaging: From Nano to Macro. Chicago, IL, pp. 849–852. 10.1109/ISBI.2011.5872537

[bib24] Dijkstra, N., Zeidman, P., Ondobaka, S., van Gerven, M. A. J., & Friston, K. (2017). Distinct Top-down and bottom-up brain connectivity during visual perception and imagery. Scientific Reports, 7(1), 5677 10.1038/s41598-017-05888-8 28720781 PMC5516016

[bib25] Estrada, E., & Hatano, N. (2008). Communicability in complex networks. Physical Revue E, 77(3 Pt 2), 036111 10.1103/PhysRevE.77.036111 18517465

[bib26] Frassle, S., Lomakina, E. I., Kasper, L., Manjaly, Z. M., Leff, A., Pruessmann, K. P., Buhmann, J. M., & Stephan, K. E. (2018). A generative model of whole-brain effective connectivity. Neuroimage, 179, 505–529. 10.1016/j.neuroimage.2018.05.058 29807151

[bib27] Friston, K., Zeidman, P., & Litvak, V. (2015). Empirical Bayes for DCM: A group inversion scheme. Frontiers in Systems Neuroscience, 9, 164 10.3389/fnsys.2015.00164 26640432 PMC4661273

[bib28] Friston, K. J. (2011). Functional and effective connectivity: A review. Brain Connectivity, 1(1), 13–36. 10.1089/brain.2011.0008 22432952

[bib29] Friston, K. J., Harrison, L., & Penny, W. (2003). Dynamic causal modelling. Neuroimage, 19(4), 1273–1302. https://doi.org/S1053811903002027 12948688 10.1016/s1053-8119(03)00202-7

[bib30] Friston, K. J., Kahan, J., Biswal, B., & Razi, A. (2014). DCM for resting state fMRI. Neuroimage, 94, 396–407. 10.1016/j.neuroimage.2013.12.009 24345387 PMC4073651

[bib31] Friston, K. J., Litvak, V., Oswal, A., Razi, A., Stephan, K. E., van Wijk, B. C., Ziegler, G., & Zeidman, P. (2016). Bayesian model reduction and empirical Bayes for group (DCM) studies. Neuroimage, 128, 413–431. 10.1016/j.neuroimage.2015.11.015 26569570 PMC4767224

[bib32] Gerraty, R. T., Davidow, J. Y., Foerde, K., Galvan, A., Bassett, D. S., & Shohamy, D. (2018). Dynamic flexibility in striatal-cortical circuits supports reinforcement learning. Journal of Neuroscience. 10.1523/jneurosci.2084-17.2018 PMC5858591 29431652

[bib33] Goni, J., Avena-Koenigsberger, A., de Mendizabal, N., van den Heuvel, M. P., Betzel, R. F., & Sporns, O. (2013). Exploring the morphospace of communication efficiency in complex networks. PLoS One, 8(3), e58070 10.1371/journal.pone.0058070 23505455 PMC3591454

[bib34] Goni, J., van den Heuvel, M. P., Avena-Koenigsberger, A., Velez de Mendizabal, N., Betzel, R. F., Griffa, A., Hagmann, P., Corominas-Murtra, B., Thiran, J. P., & Sporns, O. (2014). Resting-brain functional connectivity predicted by analytic measures of network communication. Proceedings of the National Academy of Sciences of the United States of America, 111(2), 833–838. 10.1073/pnas.1315529111 24379387 PMC3896172

[bib35] Grayson, D. S., Bliss-Moreau, E., Machado, C. J., Bennett, J., Shen, K., Grant, K. A., Fair, D. A., & Amaral, D. G. (2016). The rhesus monkey connectome predicts disrupted functional networks resulting from pharmacogenetic inactivation of the amygdala. Neuron, 91(2), 453–466. 10.1016/j.neuron.2016.06.005 27477019 PMC5233431

[bib36] Hammond, D. K., Gur, Y., & Johnson, C. R. (2013a). Graph diffusion distance: A difference measure for weighted graphs based on the graph Laplacian exponential kernel. In: 2013 IEEE Global Conference on Signal and Information Processing.Austin, TX pp. 419–422.

[bib37] Hammond, D. K., Scherrer, B., & Warfield, S. K. (2013b). Cortical graph smoothing: A novel method for exploiting DWI-derived anatomical brain connectivity to improve EEG source estimation. IEEE Transactions on Medical Imaging, 32(10), 1952–1963. 10.1109/TMI.2013.2271486 23807436 PMC3901841

[bib38] Hermundstad, A. M., Brown, K. S., Bassett, D. S., Aminoff, E. M., Frithsen, A., Johnson, A., Tipper, C. M., Miller, M. B., Grafton, S. T., & Carlson, J. M. (2014). Structurally-constrained relationships between cognitive states in the human brain. PLoS Computational Biology, 10(5), e1003591 10.1371/journal.pcbi.1003591 24830758 PMC4022461

[bib39] Honey, C. J., Kotter, R., Breakspear, M., & Sporns, O. (2007). Network structure of cerebral cortex shapes functional connectivity on multiple time scales. Proceedings of the National Academy of Sciences of the United States of America, 104(24), 10240–10245. 10.1073/pnas.0701519104 17548818 PMC1891224

[bib40] Honey, C. J., Sporns, O., Cammoun, L., Gigandet, X., Thiran, J. P., Meuli, R., & Hagmann, P. (2009). Predicting human resting-state functional connectivity from structural connectivity. Proceedings of the National Academy of Sciences of the United States of America, 106(6), 2035–2040. 10.1073/pnas.0811168106 19188601 PMC2634800

[bib41] Honey, C. J., Thivierge, J. P., & Sporns, O. (2010). Can structure predict function in the human brain? Neuroimage, 52(3), 766–776. 10.1016/j.neuroimage.2010.01.071 20116438

[bib42] Hu, C., Hua, X., Ying, J., Thompson, P. M., Fakhri, G. E., & Li, Q. (2016). Localizing sources of brain disease progression with network diffusion model. IEEE Journal of Selected Topics in Signal Processing, 10(7), 1214–1225. 10.1109/JSTSP.2016.2601695 28503250 PMC5423678

[bib43] Jenkinson, M., Bannister, P., Brady, M., & Smith, S. (2002). Improved optimization for the robust and accurate linear registration and motion correction of brain images. Neuroimage, 17(2), 825–841. 10.1006/nimg.2002.1132 12377157

[bib44] Kale, P., Zalesky, A., & Gollo, L. L. (2018). Estimating the impact of structural directionality: How reliable are undirected connectomes? Network Neuroscience, 2(2), 259–284. 30234180 10.1162/netn_a_00040 PMC6135560

[bib45] Koch, M. A., Norris, D. G., & Hund-Georgiadis, M. (2002). An investigation of functional and anatomical connectivity using magnetic resonance imaging. Neuroimage, 16(1), 241–250. 10.1006/nimg.2001.1052 11969331

[bib46] Liang, H., & Wang, H. (2017). Structure-function network mapping and its assessment via persistent homology. PLoS Computational Biology, 13(1), e1005325 10.1371/journal.pcbi.1005325 28046127 PMC5242543

[bib47] Markov, N. T., Ercsey-Ravasz, M., Van Essen, D. C., Knoblauch, K., Toroczkai, Z., & Kennedy, H. (2013). Cortical high-density counterstream architectures. Science, 342(6158), 1238406 10.1126/science.1238406 24179228 PMC3905047

[bib48] Meier, J., Tewarie, P., Hillebrand, A., Douw, L., van Dijk, B. W., Stufflebeam, S. M., & Van Mieghem, P. (2016). A mapping between structural and functional brain networks. Brain Connectivity, 6(4), 298–311. 10.1089/brain.2015.0408 26860437 PMC4939447

[bib49] Melozzi, F., Woodman, M. M., Jirsa, V. K., & Bernard, C. (2017). The virtual mouse brain: A computational neuroinformatics platform to study whole mouse brain dynamics. eNeuro, 4(3). 10.1523/ENEURO.0111-17.2017 PMC5489253 28664183

[bib50] Messe, A., Hutt, M. T., & Hilgetag, C. C. (2018). Toward a theory of coactivation patterns in excitable neural networks. PLoS Computational Biology, 14(4), e1006084 10.1371/journal.pcbi.1006084 29630592 PMC5908206

[bib51] Misic, B., Betzel, R. F., Nematzadeh, A., Goni, J., Griffa, A., Hagmann, P., Flammini, A., Ahn, Y. Y., & Sporns, O. (2015). Cooperative and competitive spreading dynamics on the human connectome. Neuron, 86(6), 1518–1529. 10.1016/j.neuron.2015.05.035 26087168

[bib52] Park, H. J., & Friston, K. (2013). Structural and functional brain networks: From connections to cognition. Science, 342(6158), 1238411 10.1126/science.1238411 24179229

[bib53] Penny, W. D. (2012). Comparing dynamic causal models using AIC, BIC and free energy. Neuroimage, 59(1), 319–330. 10.1016/j.neuroimage.2011.07.039 21864690 PMC3200437

[bib54] Pillai, A. S., & Jirsa, V. K. (2017). Symmetry breaking in space-time hierarchies shapes brain dynamics and behavior. Neuron, 94(5), 1010–1026. 10.1016/j.neuron.2017.05.013 28595045

[bib55] Pineda-Pardo, J. A., Bruna, R., Woolrich, M., Marcos, A., Nobre, A. C., Maestu, F., & Vidaurre, D. (2014). Guiding functional connectivity estimation by structural connectivity in MEG: An application to discrimination of conditions of mild cognitive impairment. Neuroimage, 101, 765–777. 10.1016/j.neuroimage.2014.08.002 25111472 PMC4312351

[bib56] Pollick, F. E., Paterson, H. M., Bruderlin, A., & Sanford, A. J. (2001). Perceiving affect from arm movement. Cognition, 82(2), B51–B61. 11716834 10.1016/s0010-0277(01)00147-0

[bib57] Proix, T., Bartolomei, F., Guye, M., & Jirsa, V. K. (2017). Individual brain structure and modelling predict seizure propagation. Brain, 140(3), 641–654. 10.1093/brain/awx004 28364550 PMC5837328

[bib58] Raj, A., Kuceyeski, A., & Weiner, M. (2012). A network diffusion model of disease progression in dementia. Neuron, 73(6), 1204–1215. 10.1016/j.neuron.2011.12.040 22445347 PMC3623298

[bib59] Razi, A., Kahan, J., Rees, G., & Friston, K. J. (2015). Construct validation of a DCM for resting state fMRI. Neuroimage, 106, 1–14. 10.1016/j.neuroimage.2014.11.027 25463471 PMC4295921

[bib60] Razi, A., Seghier, M. L., Zhou, Y., McColgan, P., Zeidman, P., Park, H. J., Sporns, O., Rees, G., & Friston, K. J. (2017). Large-scale DCMs for resting-state fMRI. Network Neuroscience, 1(3), 222–241. 10.1162/netn_a_00015 29400357 PMC5796644

[bib61] Ren, Y., Nguyen, V. T., Sonkusare, S., Lv, J., Pang, T., Guo, L., Eickhoff, S. B., Breakspear, M., & Guo, C. C. (2018). Effective connectivity of the anterior hippocampus predicts recollection confidence during natural memory retrieval. Nature Communications, 9(1), 4875 10.1038/s41467-018-07325-4 PMC6242820 30451864

[bib62] Robinson, P. A. (2012). Interrelating anatomical, effective, and functional brain connectivity using propagators and neural field theory. Physical Review E, 85(1 Pt 1), 011912 10.1103/physreve.85.011912 22400596

[bib63] Sanz Leon, P., Knock, S. A., Woodman, M. M., Domide, L., Mersmann, J., McIntosh, A. R., & Jirsa, V. (2013). The virtual brain: A simulator of primate brain network dynamics. Frontiers in Neuroinformatics, 7, 10 10.3389/fninf.2013.00010 23781198 PMC3678125

[bib64] Schirner, M., McIntosh, A. R., Jirsa, V., Deco, G., & Ritter, P. (2018). Inferring multi-scale neural mechanisms with brain network modelling. eLife, 7 10.7554/eLife.28927 PMC5802851 29308767

[bib65] Seguin, C., Razi, A., & Zalesky, A. (2019). Zalesky, A Inferring neural signalling directionality from undirected structural connectomes. Nature Communications, 10(1), 4289 10.1038/s41467-019-12201-w PMC6753104 31537787

[bib66] Seguin, C., van den Heuvel, M. P., & Zalesky, A. (2018). Navigation of brain networks. Proceedings of the National Academy of Sciences of the United States of America, 115(24), 6297–6302. 10.1073/pnas.1801351115 29848631 PMC6004443

[bib67] Senden, M., Goebel, R., & Deco, G. (2012). Structural connectivity allows for multi-threading during rest: the structure of the cortex leads to efficient alternation between resting state exploratory behavior and default mode processing. Neuroimage, 60(4), 2274–2284. 10.1016/j.neuroimage.2012.02.061 22394674

[bib68] Shen, K., Hutchison, R. M., Bezgin, G., Everling, S., & McIntosh, A. R. (2015). Network structure shapes spontaneous functional connectivity dynamics. Journal of Neuroscience, 35(14), 5579–5588. 10.1523/jneurosci.4903-14.2015 25855174 PMC6605321

[bib69] Shine, J. M., Aburn, M. J., Breakspear, M., & Poldrack, R. A. (2018). The modulation of neural gain facilitates a transition between functional segregation and integration in the brain. eLife, 7 10.7554/eLife.31130 PMC5818252 29376825

[bib70] Smith, S. M. (2002). Fast robust automated brain extraction. Human Brain Mapping, 17(3), 143–155. 10.1002/hbm.10062 12391568 PMC6871816

[bib71] Sokolov, A. A., Erb, M., Gharabaghi, A., Grodd, W., Tatagiba, M. S., & Pavlova, M. A. (2012). Biological motion processing: The left cerebellum communicates with the right superior temporal sulcus. Neuroimage, 59(3), 2824–2830. 10.1016/j.neuroimage.2011.08.039 22019860

[bib72] Sokolov, A. A., Erb, M., Grodd, W., & Pavlova, M. A. (2014). A structural loop between the cerebellum and the superior temporal sulcus: Evidence from diffusion tensor imaging. Cerebral Cortex, 24(3), 626–632. 10.1093/cercor/bhs346 23169930

[bib73] Sokolov, A. A., Kruger, S., Enck, P., Krageloh-Mann, I., & Pavlova, M. A. (2011). A gender affects body language reading. Frontiers in Psychology, 2, 16 10.3389/fpsyg.2011.00016 21713180 PMC3111255

[bib74] Sokolov, A. A., Zeidman, P., Erb, M., Ryvlin, P., Friston, K. J., & Pavlova, M. A. (2018). A structural and effective brain connectivity underlying biological motion detection. Proceedings of the National Academy of Sciences of the United States of America, 115(51), E12034–E12042. 10.1073/pnas.1812859115 30514816 PMC6305007

[bib75] Sokolov, A. A., Zeidman, P., Erb, M., Ryvlin, P., Pavlova, M. A., & Friston, K. J. (2019). Linking structural and effective brain connectivity: Structurally informed Parametric Empirical Bayes (si-PEB). Brain Structure & Function, 224(1), 205–217. 10.1007/s00429-018-1760-8 30302538 PMC6373362

[bib76] Sporns, O. (2014). Contributions and challenges for network models in cognitive neuroscience. Nature Neuroscience, 17(5), 652–660. 10.1038/nn.3690 24686784

[bib77] Sporns, O., Tononi, G., & Edelman, G. M. (2000). Theoretical neuroanatomy: Relating anatomical and functional connectivity in graphs and cortical connection matrices. Cerebral Cortex, 10(2), 127–141. 10667981 10.1093/cercor/10.2.127

[bib78] Stephan, K. E., Tittgemeyer, M., Knosche, T. R., Moran, R. J., & Friston, K. J. (2009). Tractography-based priors for dynamic causal models. Neuroimage, 47(4), 1628–1638. 10.1016/j.neuroimage.2009.05.096 19523523 PMC2728433

[bib79] Toga, A. W., Thompson, P. M., Mori, S., Amunts, K., & Zilles, K. (2006). Towards multimodal atlases of the human brain. Nature Reviews Neuroscience, 7(12), 952–966. 10.1038/nrn2012 17115077 PMC3113553

[bib80] Tzourio-Mazoyer, N., Landeau, B., Papathanassiou, D., Crivello, F., Etard, O., Delcroix, N., Mazoyer, B., & Joliot, M. (2002). Automated anatomical labeling of activations in SPM using a macroscopic anatomical parcellation of the MNI MRI single-subject brain. Neuroimage, 15(1), 273–289. 10.1006/nimg.2001.0978 11771995

[bib81] Uludag, K., & Roebroeck, A. (2014). General overview on the merits of multimodal neuroimaging data fusion. Neuroimage, 102 Pt 1, 3–10. 10.1016/j.neuroimage.2014.05.018 24845622

[bib82] Van Essen, D. C., Smith, S. M., Barch, D. M., Behrens, T. E., Yacoub, E., Ugurbil, K., & WU-Minn HCP Consortium (2013). The WU-minn Human Connectome Project: An overview. Neuroimage, 80, 62–79. 10.1016/j.neuroimage.2013.05.041 23684880 PMC3724347

[bib83] Wang, M. B., Owen, J. P., Mukherjee, P., & Raj, A. (2017). Brain network eigenmodes provide a robust and compact representation of the structural connectome in health and disease. PLoS Computational Biology, 13(6), e1005550 10.1371/journal.pcbi.1005550 28640803 PMC5480812

[bib84] Wirsich, J., Ridley, B., Besson, P., Jirsa, V., Benar, C., Ranjeva, J. P., & Guye, M. (2017). Complementary contributions of concurrent EEG and fMRI connectivity for predicting structural connectivity. Neuroimage, 161, 251–260. 10.1016/j.neuroimage.2017.08.055 28842386

[bib85] Yarkoni, T., Poldrack, R. A., Nichols, T. E., Van Essen, D. C., & Wager, T. D. (2011). Large-scale automated synthesis of human functional neuroimaging data. Nature Methods, 8(8), 665–670. 10.1038/nmeth.1635 21706013 PMC3146590

[bib86] Zeidman, P., Jafarian, A., Seghier, M. L., Litvak, V., Cagnan, H., Price, C. J., & Friston, K. J. (2019). A guide to group effective connectivity analysis, part 2: Second level analysis with PEB. Neuroimage, 200, 12–25. 10.1016/j.neuroimage.2019.06.032 31226492 PMC6711451

