# Supplementary material for: Asymmetric high-order anatomical brain connectivity sculpts effective connectivity
Source: Netw Neurosci. 2020 Sep 1;4(3):871–90. doi: 10.1162/netn_a_00150 (PMC7888488; doi:10.1162/netn_a_00150)
Supplement: Supplementary file 1 [file netn-04-871-s001.pdf]

Sokolov, A. A., Zeidman, P., Razi, A., Erb, M., Ryvlin, P., Pavlova, M. A., & Friston, Karl J. (2020). Supporting information for "Asymmetric high-order anatomical brain connectivity sculpts effective connectivity." *Network Neuroscience, 4*(3), 871-890. https://doi.org/10.1162/netn_a_00150

SUPPLEMENTARY METHODS

Dynamic causal modelling

Dynamic causal modelling is a framework for specifying differential equation models of neuronal responses, fitting these models to neuroimaging data and comparing the evidence for different models using Bayesian methods. The forward model comprises a neuronal and an observational part, where the neuronal part is as follows:

$$\dot{z} = \left(A + \sum_{w} u_w B^w\right) z + Cu \qquad \text{S1.}$$

where vector $z \in \mathbb{R}^n$ is the mass neural activity of every network node n, $\dot{z}$ is the derivative of z with respect to time, w is the experimental condition (here: happy, neutral and angry knocking stimuli) and u is a vector containing the timing of the respective experimental conditions~~is the time series of experimental input w~~ and $C \in \mathbb{R}^{n \times w}$ are the direct driving influence of each of the w experimental conditions~~inputs~~ on each network node. Extrinsic (between-node) and intrinsic (within-node) connectivity is modelled by $A \in \mathbb{R}^{n \times n}$, and parameters $B^w \in \mathbb{R}^{n \times n}$ reflect the modulatory effects of experimental manipulation w on each connection. The second, observational part of the model uses a haemodynamic model g (the extended 'Balloon' model; Stephan et al. 2007) to predict the BOLD signal y which we would expect to measure in the fMRI scanner, given the response of the neuronal model:

$$y = g(z, \theta^h) + \epsilon \qquad \text{S2.}$$

with θ^h as the parameters of the observation model and observational noise ϵ modelled as zero mean additive noise.

The estimation of DCMs is based on prior beliefs and affords posterior estimates as well as the evidence for the respective model (Friston et al. 2003). In the present study, we were

particularly interested in whether the adaptation of the prior beliefs $p(A|m)$ about the extrinsic effective connectivity $A \in \mathbb{R}^{n \times n}$ in model m according to measures of direct and indirect anatomical connectivity would contribute to optimising the model evidence $p(y|m)$, that represents the probability to observe the measured data y given model m.

According to the Bayes theorem, the posterior beliefs $p(A|y,m)$ depend on the prior beliefs $p(A|m)$ and the model evidence $p(y|m)$ in the following way:

$$p(A|y,m) = \frac{p(y|A,m)p(A|m)}{p(y|m)} \quad \text{S3.}$$

with $p(y|A,m)$ representing the likelihood distribution of the data we expect to observe given a generative model, i.e. DCM, with a certain set of parameters A. The model evidence (the denominator in Eq. S3) scores the fit of the model to the observed data:

$$p(y|m) = \iint p(y|A,m)p(A|m)\, dA \quad \text{S4.}$$

When estimating the parameters and evidence of a DCM, these integrals are approximated using variational Bayes under the Laplace approximation (Variational Laplace), as described in detail elsewhere (Friston et al. 2007). In brief, this procedure iteratively replaces a complicated posterior distribution $p(A|y,m)$ with a simpler distribution q, until their divergence is minimal. Variational Laplace also scores how far the posteriors have moved from the priors throughout the estimation process, representing model complexity.

Parametric empirical Bayes

As our analyses were conducted at the group level and DCM is a model of single-subject fMRI timeseries, we integrated structural connectivity with group level priors on effective

connectivity in PEB. PEB is a hierarchical optimisation scheme where group-level constraints (second-level posterior estimates) are iteratively applied as priors for first-level DCM inversion (estimation), thus properly accounting for within and between subject variations in effective connectivity (Friston et al. 2015). The second-level estimates are afforded by a GLM $M^{(2)}$ with the form:

$$M^{(2)}\left(A^{(2)}\right) = (X \otimes I_n)\beta \qquad \text{S5.}$$

with $X \in \mathbb{R}^{S\times C}$ as the design matrix containing subjects S and covariates C, I_n the identity matrix of dimension N (number of A parameters in the DCM) and $\otimes$ as operator duplicating each element of X for each parameter A. The parameters of the GLM are $\beta \in \mathbb{R}^{CN\times 1} \subset A^{(2)}$ that, upon estimation, serve as empirical priors for first-level DCM re-estimation and/or analytical inference on model evidence and posterior parameters using BMR (Friston et al. 2016). In other words, we summarise the effective connectivity in terms of a posterior density over the group mean.

Bayesian model reduction

Having estimated a 'full' DCM or PEB model containing all parameters of interest, BMR can be used for analytical derivation of the parameters and evidence for reduced models, i.e. models with less or adapted parameters (Rosa et al. 2012; Friston et al. 2016). Contingent upon the availability of a full model's evidence $p(y|m_F)$ and posterior parameter distribution $p(A|y, m_F)$, reformulation of Eq. S4 indicates that the comparison of modified (reduced) priors $p(A|m_R)$ against the full priors $p(A|m_F)$ of the estimated model can directly yield the reduced model evidence $p(A|y, m_R)$:

$$p(A|y,m_R) = p(A|y,m_F)\frac{p(y|m_F)p(A|m_R)}{p(y|m_R)p(A|m_F)}$$

$$\frac{p(y|m_R)}{p(y|m_F)} = \int p(A|y,m_F)\frac{p(A|m_R)}{p(A|m_F)}dA$$

S6.

REFERENCES

Friston K, Mattout J, Trujillo-Barreto N, Ashburner J, Penny W (2007). Variational free energy and the Laplace approximation. *Neuroimage 34*(1), 220-234. DOI: 10.1016/j.neuroimage.2006.08.035

Friston K, Zeidman P, Litvak V (2015). Empirical Bayes for DCM: A Group Inversion Scheme. *Front Syst Neurosci 9*(164. DOI: 10.3389/fnsys.2015.00164

Friston KJ, Harrison L, Penny W (2003). Dynamic causal modelling. *Neuroimage 19*(4), 1273-1302. DOI: S1053811903002027 [pii]

Friston KJ, Litvak V, Oswal A, Razi A, Stephan KE, van Wijk BC, Ziegler G, Zeidman P (2016). Bayesian model reduction and empirical Bayes for group (DCM) studies. *Neuroimage 128*(413-431. DOI: 10.1016/j.neuroimage.2015.11.015

Rosa MJ, Friston K, Penny W (2012). Post-hoc selection of dynamic causal models. *J Neurosci Methods 208*(1), 66-78. DOI: 10.1016/j.jneumeth.2012.04.013

Stephan KE, Weiskopf N, Drysdale PM, Robinson PA, Friston KJ (2007). Comparing hemodynamic models with DCM. *Neuroimage 38*(3), 387-401. DOI: 10.1016/j.neuroimage.2007.07.040
