# Supplementary figures and images for: Asymmetric high-order anatomical brain connectivity sculpts effective connectivity
Source: Netw Neurosci. 2020 Sep 1;4(3):871–90. doi: 10.1162/netn_a_00150 (PMC7888488; doi:10.1162/netn_a_00150)

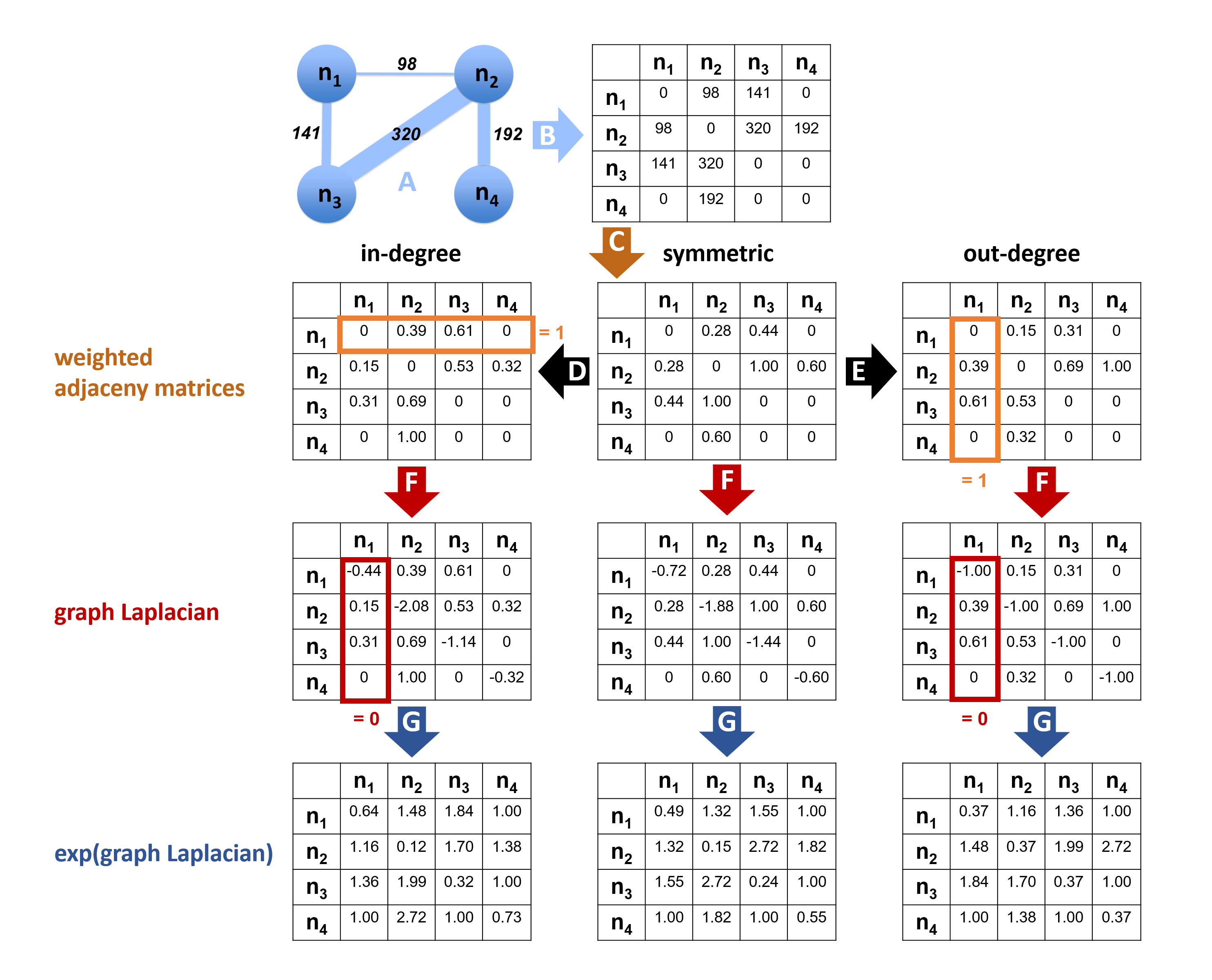

Supplement: Supplementary file 2 [file netn-04-871-s002.tif]

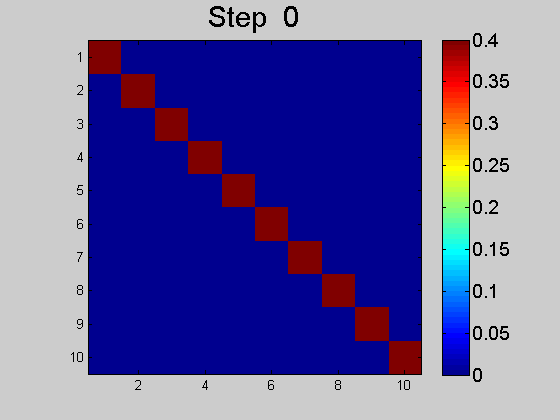

Supplement: Supplementary file 3 [file netn-04-871-s003.gif]
